# Physical and Mental Health in Adolescence: Novel Insights from a transdiagnostic examination of FitBit data in the ABCD Study

**DOI:** 10.21203/rs.3.rs-3270112/v1

**Published:** 2023-10-03

**Authors:** Katherine Damme, Teresa Vargas, Sebastian Walther, Stewart Shankman, Vijay Mittal

**Affiliations:** Northwestern University; Northwestern University; University of Bern; Northwestern University; Northwestern University

## Abstract

Adolescence is among the most vulnerable period for the emergence of serious mental illnesses. Addressing this vulnerability has generated interest in identifying markers of risk for symptoms and opportunities for early intervention. Physical fitness has been linked to psychopathology and may be a useful risk marker and target for early intervention. New wearable technology has made assessing fitness behavior more practical while avoiding recall and self-report bias. Still, questions remain regarding the clinical utility of physical fitness metrics for mental health, both transdiagnostically and along specific symptom dimensions. The current study includes 5007 adolescents (ages 10 to 13) who participated in the Adolescent Brain Cognitive Development (ABCD) study and additional sub-study that collected fitness data from wearable technology and clinical symptom measures. Physical fitness metrics included resting heart rate (RHR- an index of cardiovascular health), time spent sedentary (associated with increased inflammation and cardiovascular disease), and time spent in moderate physical activity (associated with increased neurogenesis, neuroplasticity, and healthy neurodevelopment). Self-report clinical symptoms included measures of internalizing symptoms, externalizing symptoms, and psychosis-like experiences - PLE). Increased RHR- lower cardiovascular fitness- related only to greater internalizing symptoms (t = 3.63). More sedentary behavior related to elevated PLE severity (t = 5.49). More moderate activity related to lower PLE (t=−2.69) and internalizing (t=−6.29) symptom severity. Wearable technology fitness metrics linked physical health to specific mental health dimensions, which emphasizes the utility of detailed digital health data as a marker for risk and the need for precision in targeting physical health behaviors to benefit symptoms of psychopathology.

## Introduction

Adolescence is a critical window in which symptoms of psychopathology tend to emerge.^1–4^ This period is also a critical developmental window defined by marked changes in metabolic, hormonal, neural, and social health, which have a major impact on physical and mental health.^1,5^ These features make adolescence an ideal period for interventions that may promote physical and mental health. Addressing health behaviors may be an important treatment target as sedentary behavior is increasing and physical activity is decreasing among adolescents both due to an increasingly digital world and in the aftermath of the global pandemic.^6^ Although reviews of physical activity in adolescence demonstrate a benefit to metabolic, neural, and mental health outcomes, these findings depend on recall and self-report data^1–4^ that may be distorted by symptoms of psychopathology.^7–9^ The recent expansion of wearable technology^10,11^ has improved the practicality of indexing actual health behaviors of individuals.^1, 12–15^ Cardiovascular fitness measures, such as resting heart rate, previously required in-lab assessment but can now reliably be assessed with wearable technology.^10,11^ Additionally, few existing studies include more than one mental health symptom dimension. As a result, it is unclear whether fitness behaviors protect against emerging psychopathology generally, or are associated with specific symptom dimensions of mental illness (i.e., psychosis-like experiences (PLEs), internalizing, externalizing domains).

Physical health behaviors have been associated with mental health outcomes^2, 16–18^ in parallel studies of psychosis spectrum, internalizing, and externalizing psychopathologies.^12–15, 19–22^ Across these clinically siloed studies, lower cardiovascular fitness^2,7,23,24^ and more time spent in sedentary behavior^24–29^ have been associated with symptoms of psychosis and internalizing psychopathologies (especially depression and anxiety). Time spent engaged in moderate to intense physical activity has been related to lowered symptoms across diagnoses of psychosis,^24^ internalizing,^18^ and externalizing psychopathologies both in observational^2, 16–18^ and intervention^12–15, 19–22^ studies. Similar observations across parallel clinical literatures may suggest a broad clinical benefit to improving physical activity regardless of symptom dimension. This transdiagnostic benefit would be of great practical utility in early adolescence when symptoms of psychopathology begin to emerge but have not necessarily differentiated individuals into specific diagnostic categories. However, each health metric may have unique contributors to mental health symptoms.^30^ Indeed, Stubbs et al. (2017) showed that cardiovascular health, sedentary behaviors, and physical activity (number of steps) have unique contributions to psychosis symptoms through distinct metabolic pathways. This specificity may also provide insights into potential disease mechanisms that may have relevance to the risk for or treatment of disorders.

In studies of adolescence, physical fitness metrics are related to an array of outcomes.^1,31^ Prior Adolescent Brain Cognitive Development ^®^ (ABCD) studies found that more optimal body mass index or body morphometry related to better health,^5,32,33^ cognitive outcomes,^33–35^ and brain development.^5,34,35^ Although adolescent work has linked fitness metrics to larger categories of psychopathologies (PLEs, internalizing, externalizing), these symptom dimensions are often examined in parallel^2,13^ and do not account for the symptoms within the same individuals.^3,4^ Indeed, previous ABCD work has examined a smaller baseline subsample of the fitness data (particularly resting heart rate and the number of daily steps), finding that decreased fitness related to increased internalizing symptoms.^9^ Questions remain regarding the transdiagnostic or symptom-specific benefit of health behaviors, which is ultimately necessary for targeted intervention and prevention efforts. If physical health relates to mental health transdiagnostically, then fitness could be used broadly as a potential risk marker or treatment target. In contrast, specificity in this relationship would emphasize a need for clinical precision when using physical health as a marker or treatment target for mental health outcomes.

The current paper examines the relationship between real-world physical fitness metrics (resting heart rate, time spent sedentary, time spent engaged in moderate to intense physical activity) recorded over several days for multiple weeks and clinical symptom dimensions in the ABCD Study. These analyses take advantage of a large, diverse national cohort^36^ with real-world fitness and fitness behavior metrics from publicly available wearable technology.^10,11^ This sample does not rely on self-reported fitness behaviors or short-term health and behavior lab assessments.^9,11,34,35^ Instead, this rich dataset allows us to draw on a wider range of data in the largest characterization of physical health and mental health symptom dimensions.^37^ We expect poor cardiovascular fitness (higher resting heart rate) will be associated with poor mental health based on prior self-report and descriptive actigraphy studies.^1,2,24,26^ Greater time spent sedentary, we expect to be associated with more PLEs and internalizing symptoms.^24–29^ Finally, we hypothesize that more time spent in moderate-to-intense physical activity will be associated with better mental health across symptom dimensions.^2,13,14,18,19,23,38^

## Methods

### Participants.

The ABCD Study included 21 sites across the United States, recruiting participants (aged 10–13 years) with a broad demographic diversity range. The current subsample included 5007 individuals who participated in the regular clinical symptom assessment and the optional Fitbit portion of the study acquired during year 2 of data collection. This subsample ranged in age from 10.58 to 13.5 years, was 48.41% female, and included a wide racial and socioeconomic makeup, Table 1. Features of the sample were included in the model, including sex assigned at birth (male/female), age (in months), socioeconomic status (category), and body mass index (kg/m^2^), due to their known association with cardiovascular health. All research protocols were approved by each respective institutional review board, including obtaining parents’ informed consent and child’s assent.

#### Prodromal Questionnaire-Brief Child Version (PQ-CB).

PQ-CB is a 21-item self-report questionnaire answered by the participant.^39^ Each item is endorsed as present or not and then rated on a distress scale on a 6-point scale (0 indicates not present; 1 indicates no distress; 2–6 indicates present and some level of distress). The symptom severity score is a total of the distress (only items scored 2–6 in terms of distress) for all 21 items; items without a distress score of at least 2 were treated as 0 (not present), consistent with previous scoring and validations.^39,40^

#### Child Behavioral Checklist (CBCL).

Participants used an automated self-report version of the CBCL.^41–43^ The CBCL is a 113-item questionnaire that measures aspects of the participants’ behavior across the past six months; each item was rated on a 3-point scale (not true, somewhat, or sometimes true, very or often true). Responses were used to generate ABCD-curated total scores for internalizing (including depression and anxiety subscales) and externalizing symptoms, which have been previously validated.^9, 41–43^ Only those individuals who responded to every item were included in the analyses. Follow-up analyses included raw symptom scores which did not change the magnitude or direction of the effects from t-score assessments.

### Fitness Assessments.

Fitbit Charge HR2 devices used proprietary algorithms to measure health metrics at a one-second sampling rate for heart rate using photoplethysmography, which has been validated against gold-standard measures of heart rate.^9, 44–46^ Heart rate metrics were used to calculate resting heart rate, minutes spent in sedentary activity, and minutes spent in moderate to intense activity (a total of minutes spent fairly active and very active). The ABCD Study calculated fitness metrics by week and provided filtering standards for data that met the minimum quality. Curated weekly fitness summaries were acquired for individuals with at least 600 minutes of continuous data per day for at least four weekdays and at least 1 weekend day for three weeks (*M* = 874.3, *StD* = 66.6 per day). For all tness metrics, a grand mean was created across the days of the week, which were then additionally averaged across all the weeks for which data was available. Follow-up analyses included a correction for the average number of daily hours with data (Supplemental Table 1) and a proportion of recorded time spent in sedentary or moderate activity^47^ (Supplemental Table 2), which did not change the magnitude or direction of reported findings. Finally, the moderate to intense activity level included a sum of the time spent active and very active based on previous research.^47^

### Analytical Strategy.

For each FitBit Metric, separate multilevel models examined the relationship between a single fitness metric (resting heart rate, time spent sedentary, time spent in moderate to intense activity) with all three of the symptom dimensions (psychosis-like experience symptoms severity (PLE), internalizing, externalizing symptoms), accounting for the random effects of familial relatedness, and the fixed effects of sex, age, socioeconomic status, and body mass index using lme4 in Rv.4.2.1. Composite symptoms relating to fitness were explored for specificity within the symptom domain (e.g., anxiety and depression subdomains for the internalizing composite symptoms). These additional analyses were done only in the cases of significant effects to reduce the overall number of models tested. Follow-up analyses created a natural log transformation of the symptom scores to address the high number of zero symptom scores, which did not alter the interpretation, magnitude, or direction of the reported effects. For replicability and transparency, all analytic code has been provided.^51^ Although all effect sizes will be provided, only those effects that pass correction for multiple comparisons for three fitness metrics (Bonferroni correction *p* = .017) will be interpreted. All model parameters were largely independent (intercorrelation of fixed effects r’s < .10; Variable Inflation Factor < 3.48).

## Results

### Participants.

Participants’ demographic descriptions and model parameters are available in Table 1.

### Fitness metrics.

**Figure 1:**
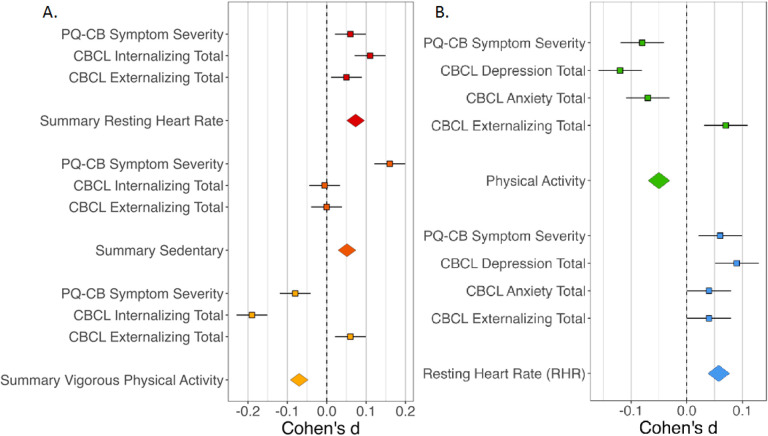
Fitness Metric Effect by Symptom Dimension: A. Symptom Composite Model B. Follow-Up Symptom Subscale Model; Error bar reflects standard error; PQ-CB - Prodromal Questionnaire-Brief Child Version; CBCL – Child Behavioral Checklist

Fitness metrics were predicted by current symptoms (PLEs, internalizing, externalizing) in a multilevel model that accounted for the random effects of individuals and relatedness and the fixed effects of age, sex, socioeconomic status, and body mass index, Fig. 1A. Full statistics for all model parameters are provided in Table 2.

### Psychosis-Like Experience Severity.

**Figure 2:**
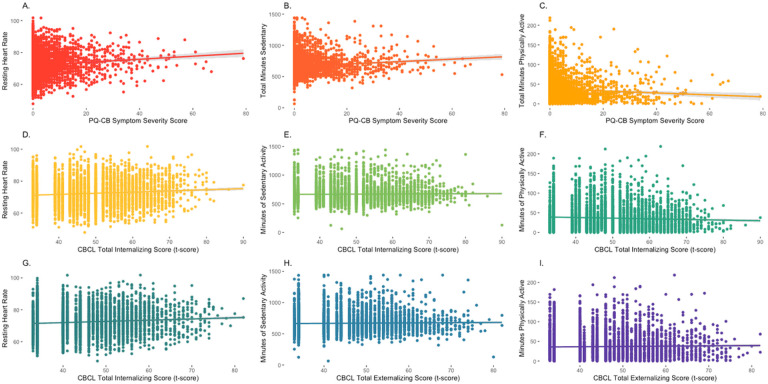
Current Symptoms Related to Fitness Metrics; PQ-CB - Prodromal Questionnaire-Brief Child Version; CBCL – Child Behavioral Checklist

There was a main effect of current PLE severity on resting heart rate, *t*(4542) = 2.13, p = .033, which did not survive correction for multiple comparisons, Fig. 2A. There was a significant main effect of current PLE severity on time spent sedentary, *t*(4514) = 5.49, *p* < .001, such that greater current PLE severity related to more time spent sedentary, Fig. 2B. There was a significant main effect of current PLE severity on time spent in moderate to intense physical activity, *t*(4522)=-2.70, p = .007, such that less current PLE severity related to more time spent physically active, Fig. 2C.

### Internalizing Total Symptoms.

There was a significant main effect of current total internalizing symptoms on resting heart rate, *t*(4536) = 3.63, *p* = .0002, such that greater current internalizing symptoms were related to elevated resting heart rate (Fig. 2D). There was no significant main effect of current total internalizing symptoms on time spent sedentary, *t*(4520)= −0.18, *p* = .86; Fig. 2E. There was a significant main effect of current total internalizing symptoms on time spent in moderate physical activity, *t*(4513)=−6.286, *p* < .001, such that less current total internalizing symptoms were related to more time in moderate physical activity, Fig. 2F.

### Externalizing Total Symptoms.

There was no significant main effect of current total externalizing symptoms on resting heart rate, *t*(4484) = 1.76, *p* = .08, Fig. 2G. There was no significant main effect of current total externalizing symptoms on time spent sedentary, *t*(4435) = 0.81, *p* = .99, Fig. 2H. There was no significant main effect of current total externalizing symptoms on time spent in moderate activity, *t*(4448) = 2.159, *p* = .03, Fig. 2I.

### Follow-Up Analyses of Anxiety and Depression Dimensions.

Follow-up analyses were conducted for only those fitness metrics that were significantly related to internalizing symptoms (resting heart rate and moderate to intense physical exercise; Bonferroni correction adjusted for two models). Metrics were predicted by current symptoms in a multilevel model that accounted for the random effects of individuals and relatedness and the fixed effects of age, sex, socioeconomic status, and body mass index. Full statistics for all model parameters are provided in Table 3; effects by symptom dimension are depicted in Fig. 1B.

There was a significant main effect of current total depression symptoms on resting heart rate, *t*(4445) = 3.073, *p* = .002, such that greater current depression symptoms were related to elevated resting heart rate. There was a significant main effect of current total depression symptoms on the total time spent engaged in moderate to intense physical activity, *t*(4403)=−4.043, *p* < .001, such that less current depression symptoms were related to more time spent engaged in moderate to intense physical activity. There was no significant main effect of anxiety disorder symptoms on resting heart rate, *t*(4391) = 1.45, *p* = .15. There was a significant main effect of current total anxiety symptoms on the total time spent engaged in moderate to intense physical activity, *t*(4337)=−2.392, *p* = .017, such that less current anxiety symptoms were related to more time spent engaged in moderate to intense physical activity.

## Discussion

Wearable technology metrics of fitness and fitness behaviors showed unique patterns of relationships to mental health dimensions. This study strengthens the evidence for a clear link between physical and mental health.^2,31,38,48^ Despite expectations that physical health would relate to lower overall psychopathology, physical health behaviors showed specificity among mental symptom dimensions. Better cardiovascular health (lower resting heart rate) was related to lower internalizing symptoms, which were driven by depression symptoms and not anxiety. In contrast, increased time spent sedentary had unique relevance to PLE severity but not internalizing or externalizing symptoms. Finally, greater time spent engaged in moderate to intense physical activity was related to lower levels of both PLE severity and internalizing symptoms, but not externalizing symptoms. Features of physical health are related to dimensions of mental health, but clinical precision may be required when using physical health as a marker of risk or target for mental health intervention to match the right target to the right symptoms.

Cardiovascular health (resting heart rate) was related to current internalizing symptoms in adolescence. This finding extends a large, decades-long literature linking depression^2,16,49^ in three ways: moving findings earlier in development, examining longer real-world assessments of resting heart rate, and accounting for additional symptom dimensions within an individual. Cardiovascular health was related to fewer internalizing symptoms (d=−0.19), which was driven by fewer depression symptoms (d=−0.17) rather than anxiety (d = .07). Though this effect size is small, it is similar to previously reported effects (d=-0.166).^4^ Despite previous literature suggesting a general relationship between cardiovascular health and mental health across the psychosis spectrum^7,23,24,26,50,51^ and internalizing,^2–4, 18,20,21,52^ the current study found that internalizing alone was related when accounting for other symptom dimensions within the model. However, it is noteworthy that PLE severity and internalizing symptom effects were in a similar direction and magnitude to the noted findings, albeit not significant. These findings emphasize the need for future work to examine the relationship between multiple symptom dimensions to explore this specificity.

Increased time spent sedentary was related to greater PLEs severity. This finding is consistent with many studies demonstrating that symptoms across the psychosis spectrum are related to greater time spent sedentary.^24,25,27,28,47^ These findings highlight the potential for time spent sedentary to be explored as a relevant early risk factor for psychosis that shows specificity among other symptom dimensions. Additionally, the specificity of this behavior may merit additional investigations into biological mechanisms related to increased sedentary behaviors (e.g., increased in ammation)^25^ as potential mechanisms of emerging PLE severity.

More time spent engaged in moderate physical activity related to reduced PLE severity and internalizing symptoms, but not externalizing symptoms. This finding is consistent with a growing literature suggesting a beneficial impact of physical exercise on attenuated and clinical psychosis symptoms.^16,23^ It was expected that physical activity would generally benefit all mental health dimensions. In contrast, moderate to intense physical activity may only relate to PLE severity and internalizing disorders and may not be associated with externalizing disorders, which highlights the importance of considering symptom dimensions for targeted interventions as well as risk behaviors.

Despite several strengths of this study, but it is also important to note relevant limitations and future directions. First, these individuals are young and relatively healthy, which may limit the range of physical and mental health impacts observed in the current sample.^53^ This preclinical range is also a strength, as the current findings are more generalizable to preventative interventions in adolescent populations. This relatively healthy sample may also underestimate the relationship between physical and mental health. Although the reported effects were small (*d* = .08–.19), they are similar to other risk markers of psychopathology, including paternal diagnoses (*d* = 0.21),^54^ risk genes (*d* = 0.096–.17),^55^ and irritable temperament (*d* = 0.16).^54^ Next, prior intervention studies demonstrated that physical activity is an effective intervention for reducing externalizing symptoms.^3,14,22,38^ Reductions in externalizing symptoms may be related to the change in dosage of physical activity level, which would not appear from the current observational approach.^22,38^ Further, some previous studies observed that increased physical activity related to increased externalizing psychopathologies.^4,48^ Additionally, it is notable that the current metrics reflect aggregate measures but follow-up analyses suggested that the amount of time did not bias the primary findings (See Supplemental Information). Furthermore, causality cannot be determined in the current sample; more research is needed to elucidate whether changes in health behavior precede changes in symptoms, making health data a useful risk marker that could potentially be modified by targeted interventions. Finally, future studies should consider examining the relationship to specific symptoms, e.g., motor slowing^56–58^, coordination,^28^ dysphoria,^3,22^ that may provide new insight into the types of patients that may benefit most from exercise interventions.

There are several strengths of this study. First, the study accounts for co-occurring dimensions of clinical symptoms within the same individuals. This model approach allows us to directly compare the relationship of health metrics to relevant symptom dimensions while accounting for levels of psychopathology along other dimensions. Second, the study reflects a large, diverse cohort of adolescents; the size and diversity of this cohort provide confidence in the estimating of the effects and the generalizability of the findings. Finally, the study was in emerging adolescence, a time when psychopathology typically emerges. As a result, these relationships have strong implications for the relevance of exercise as an early and preventative intervention. Taken together, these findings may suggest that physical activity shows a dimension-specific reduction in symptoms. The current investigation demonstrates this relationship between physical and mental health in a pre-adolescence sample, immediately preceding the emergence of mental illness diagnoses and unmasking vulnerability. It is also notable that this early adolescent population has experienced fewer of the impacts that mental health issues have on physical health, e.g., impact of medication and neurotoxic effects of mental health episodes^53^. Similarly, individuals experiencing mental health issues also may have their fitness activities impacted by changes in mental health. As a result, this study strengthens the evidence for a clear link between physical and mental health^2,31,38,48^. That this relation becomes apparent during this pre-adolescence period has possibly sizable implications for earlier intervention and prevention efforts^17,59^. Finally, these results are somewhat surprising as a growing body of evidence has targeted health behaviors to improve mental health diagnoses in parallel. However, the specificity of particular digital health metrics to particular symptoms highlights an opportunity to tailor these interventions to address the specific symptoms and needs of an individual.

## Figures and Tables

**Table 1:** Fitness Metrics ABCD Subsample Demographics

Demographic Parameter	Mean (StD)
Age at Interview	11.95 (0.65)
BMI	20.17 (4.32)
Sex at Birth	%
Female	48.41%
Male	51.59%
Race	%
Asian	2.40%
Black	9.47%
Mixed	11.38%
Other	3.87%
White	72.21%
AIAN/NHPI	0.67%
Household Income	%
<50K	23.18%
>=50K & <100K	30.98%
>=100K	45.83%

**Table 2:** Multilevel Model of Fitness Metrics to Current Symptoms with all parameters

Fitness Metric	Parameter	t-value	p-value	Bonferroni
Resting Heart Rate (RHR)	PLE Severity	2.127	3.30E-02	
	Internalizing Symptoms	3.632	2.84E-04	*
	Externalizing Symptoms	1.758	7.88E-02	
	Age at Interview	−9.649	< 2e-16	*
	Sex at Birth	−12.391	< 2e-16	*
	BMI	12.723	< 2e-16	*
	Income ( > = 100k vs > 50k)	−5.967	2.62E-09	*
	Income (50k–100k vs > 50k)	−2.427	0.015278	*
Sedentary Activity	PLE Severity	5.492	4.20E-08	*
	Internalizing Symptoms	−0.182	8.55E-01	
	Externalizing Symptoms	−0.813	0.99	
	Age at Interview	7.911	3.23E-15	*
	Sex at Birth	1.558	1.19E-01	
	BMI	6.477	1.03E-10	*
	Income ( > = 100k vs > 50k)	−5.804	6.96E-09	*
	Income (50k–100k vs > 50k)	−4.114	3.96E-05	*
Moderate Activity	PLE Severity	−2.698	7.01E-03	*
	Internalizing Symptoms	−6.286	3.57E-10	*
	Externalizing Symptoms	2.159	0.03088	
	Age at Interview	0.238	0.81155	
	Sex at Birth	32.828	< 2e-16	*
	BMI	18.517	< 2e-16	*
	Income ( > = 100k vs > 50k)	6.91	5.60E-12	*

**Table 3:** Multilevel Model of Fitness Metrics to Current Symptoms With Depression and Anxiety Subtotals with all parameters

Fitness Metric	Parameter	t-value	p-value	Bonferroni
Resting Heart Rate (RHR)	PLE Severity	1.923	5.46E-02	
	Depression Symptoms	3.073	2.13E-03	*
	Anxiety Symptoms	1.451	.147	
	Externalizing Symptoms	1.324	0.18571	
	Age at Interview	−9.662	< 2e-16	*
	Sex at Birth	−12.417	< 2e-16	*
	BMI	12.7	< 2e-16	*
	Income ( > = 100k vs > 50k)	−5.832	5.91E-09	*
	Income (50k–100k vs > 50k)	−2.28	0.02267	*
Moderate to Intense Physical Activity	PLE Severity	−2.506	1.23E-02	*
	Depression Symptoms	−4.043	5.37E-05	*
	Anxiety Symptoms	−2.392	0.01678	*
	Externalizing Symptoms	2.145	0.03201	
	Age at Interview	0.36	0.71851	
	Sex at Birth	32.854	< 2e-16	*
	BMI	18.498	< 2e-16	*
	Income ( > = 100k vs > 50k)	6.675	2.82E-11	*
	Income (50k–100k vs > 50k)	2.632	0.00852	*
